# Clinical Characteristics of *Mycoplasma pneumoniae* Pneumonia in Children and Risk Factors for Progression to Severe Disease

**DOI:** 10.1155/carj/5938199

**Published:** 2026-09-15

**Authors:** Chao Fu, Xiang He, Chengcheng Li, Shirong Luo, Yunrong Li

**Affiliations:** ^1^ The First People’s Hospital of Zunyi (The Third Affiliated Hospital of Zunyi Medical University), Zunyi Guizhou, 563000, China

**Keywords:** children, *Mycoplasma pneumoniae*, pneumonia, risk factors, severe

## Abstract

**Objective:**

To understand the clinical characteristics of severe and nonsevere *Mycoplasma pneumoniae* pneumonia (MPP) in children and analyze the risk factors for severe *Mycoplasma pneumoniae* pneumonia (SMPP), providing a basis for early clinical diagnosis and treatment of SMPP.

**Methods:**

A retrospective analysis was conducted on 146 children with MPP admitted to pediatric wards 1–4 of Zunyi First People’s Hospital from August 2024 to June 2025. According to the severity of their condition, the patients were divided into a severe group (*n* = 49) and a nonsevere group (*n* = 97). Clinical manifestations, imaging data, and laboratory indicators were compared between the two groups, and logistic regression analysis was used to summarize the risk factors for pediatric SMPP.

**Results:**

(1) The duration of fever and cough was significantly longer in the severe MPP group than in the nonsevere group (*p* < 0.05). Compared with those in the nonsevere group, instances of prematurity, intrauterine infection at birth, pulmonary consolidation, pleural effusion, high fever, wheezing, and difficulty breathing were significantly more frequent in the severe group (*p* < 0.05). (2) Laboratory indicators, such as C‐reactive protein levels, lactate dehydrogenase levels, D‐dimer levels, ferritin levels, the neutrophil ratio, and serum amyloid A levels, were significantly greater in the severe group than in the nonsevere group (*p* < 0.05). (3) The risk factors for SMPP included prematurity (OR = 4.747; 95% CI: 1.259 ∼ 17.891; *p* = 0.021), pulmonary consolidation (OR = 5.277; 95% CI: 1.372 ∼ 20.301; *p* = 0.016), high fever (OR = 25.597; 95% CI: 6.760 ∼ 96.926; *p* < 0.001), wheezing (OR = 6.913; 95% CI: 1.837 ∼ 26.017; *p* = 0.004), and difficulty breathing (OR = 5.990; 95% CI: 1.451 ∼ 24.722; *p* = 0.013).

**Conclusion:**

Children with SMPP have prolonged fever and cough, more severe clinical symptoms, and significantly elevated inflammation‐related laboratory indicators. Prematurity, pulmonary consolidation, high fever, wheezing, and difficulty breathing are easily accessible baseline warning indicators for early identification of children at high risk of progression to SMPP. Strengthening early recognition and timely intervention in patients presenting with these factors may help optimize clinical management and improve outcomes.

## 1. Introduction

*Mycoplasma pneumoniae* (MP) is among the most important pathogens that causes community‐acquired pneumonia (CAP) in children. In different regions, the detection rate of CAP among children over 5 years old can reach 10%–40%, and it is prone to periodic epidemics [1–3]. In recent years, with the widespread adoption of molecular biology detection techniques, the incidence of *Mycoplasma pneumoniae* pneumonia (MPP) has gradually increased, and in some areas, it has become the leading pathogen of pediatric CAP [4, 5]. Although most cases are mild to moderate in severity, a significant proportion of children still present with severe or even refractory pneumonia in clinical practice, leading to prolonged hospitalization, increased complications, and even life‐threatening conditions [6, 7].

The clinical manifestations of pediatric MPP lack specificity, typically presenting as persistent fever, refractory cough, and pulmonary imaging changes, but there is also some heterogeneity among different children [8, 9]. In recent years, the factors influencing the severity of MPP have gradually gained attention. Studies indicate that age, host immune status, mode of delivery, season of onset, and abnormal laboratory indicators may all be associated with the severity and prognosis of MPP [10]. Additionally, the emergence of drug‐resistant strains of MP has led to prolonged disease courses in some children, increasing treatment difficulty [11, 12]. However, current systematic research on the clinical characteristics and risk factors for pediatric MPP remains insufficient, with varying results across different regions and populations, necessitating more clinical data support [13, 14].

Given this background, this study conducted a retrospective analysis of pediatric MPP patients admitted to Zunyi First People’s Hospital, summarizing their clinical characteristics and exploring risk factors related to disease severity, aiming to provide clinical evidence for early identification of high‐risk children, optimization of treatment strategies, and improvement of prognosis.

## 2. Materials and Methods

### 2.1. Study Population

In this retrospective study, 146 children with MPP admitted to the Department of Pediatrics at Zunyi First People’s Hospital from August 2024 to June 2025 were selected. The patients were divided into the SMPP group (*n* = 49) and the general MPP group (*n* = 97) on the basis of disease severity.

### 2.2. Ethical Statement

This study was conducted in accordance with the Declaration of Helsinki and approved by the Medical Ethics Committee of Zunyi First People’s Hospital (Ethical Review (2023)‐1‐184). The parents of the children involved in this study were informed and signed informed consent forms. It should be noted that this study poses no risk to the children, and data on the disease course and children’s examinations were obtained through the hospital’s electronic medical record system.

### 2.3. Diagnostic Criteria

#### 2.3.1. Diagnostic Criteria for MPP

In accordance with the *Guidelines for the Diagnosis and Treatment of Mycoplasma pneumoniae Pneumonia in Children (2023) Edition,* the diagnostic criteria for MPP include the following: (1) Clinical Manifestations: Fever and cough are the main clinical manifestations, which may be accompanied by headache, runny nose, sore throat, ear pain, etc. Fever is mainly moderate to high grade, and persistent high fever indicates severe illness. The cough is intense, resembling whooping cough, and is mostly dry in the early stages. Some children may exhibit wheezing. (2) Physical Examination: Early pulmonary signs may be subtle, but as the disease progresses, decreased breath sounds and dry or wet rales may appear. (3) Imaging Findings: Early chest X‐rays or CT scans of MPP primarily show thickening and increased bronchovascular markings, thickened bronchial walls, possible ground‐glass opacities, “tree‐in‐bud” signs, interlobular septal thickening, and reticular shadows. Alveolar inflammatory changes vary depending on the extent of alveolar involvement, presenting as ground‐glass opacities and patchy, segmental, or even lobar consolidations, often with atelectasis, and may be accompanied by hilar lymphadenopathy. Severe cases may involve pleural effusion. Unilateral involvement is more common than bilateral involvement, and lesions may or may not show air bronchograms. Consolidations appear as moderate‐ to high‐density shadows, with higher density correlating with larger consolidation areas and more affected lobes. Lesions of varying shapes, sizes, and densities may coexist. Mucoid impaction signs may also be present. Some MPP patients may exhibit localized or diffuse bronchiolitis features, with high‐resolution CT (HRCT) showing centrilobular nodules, “tree‐in‐bud” signs, branching linear opacities, bronchiolar dilation, and mosaic patterns, as well as bronchial inflammation manifesting as thickened bronchial walls and mucus plugging. When MPP is complicated by pulmonary complications, such as pulmonary embolism (PE) or necrotizing pneumonia (NP), corresponding imaging changes may occur (see Complications). 4) Etiological or Serological Findings: Nucleic Acid Testing: Molecular diagnostics for DNA or RNA are positive upon infection, offering high sensitivity and specificity, making it the most critical method for early diagnosis. Serum IgM Antibody Testing: This can serve as a diagnostic basis. MP‐IgM antibodies typically appear 5–7 days postinfection, rendering this method unsuitable for early diagnosis within the first 5 days of illness or for immunocompromised patients with MPP requiring early etiological diagnosis.

The diagnosis of MPP can be established if the clinical and imaging manifestations mentioned above are present along with any one or both of the following criteria: (1) Positive MP‐DNA or RNA. (2) A single serum MP antibody titer ≥ 1:160 (PA method) or a fourfold or greater increase in the MP antibody titer between paired serum samples during the course of the illness.

#### 2.3.2. Diagnostic Criteria for SMPP

In accordance with the *Guidelines for the Diagnosis and Treatment of Mycoplasma pneumoniae Pneumonia in Children (2023 Edition)* [15], the diagnostic criteria for SMPP include meeting any one of the following manifestations: (1) Persistent high fever (above 39°C) ≥ 5 days or fever ≥ 7 days, with no decrease or increase in peak body temperature, accompanied by high fever; (2) the presence of wheezing, shortness of breath, difficulty breathing, chest pain, hemoptysis, or any one of these symptoms. These manifestations are associated with severe lesions, combined plastic bronchitis, asthma exacerbation, pleural effusion, and PE; (3) occurrence of extrapulmonary organ complications that do not meet critical illness criteria; (4) resting oxygen saturation ≤ 0.93 at sea level while breathing room air; (5) imaging findings revealed any one of the following: (1) high‐density lung consolidation involving more than approximately two‐thirds of one lobe or high‐density consolidation in two or more lobes (regardless of the extent of involvement); (2) unilateral or bilateral diffuse bronchiolitis manifestations, which may be combined with bronchitis (bronchial wall thickening, peribronchial exudation, and possible mucus blockage leading to atelectasis). 6. Progressive clinical worsening with imaging showing lesion progression exceeding 50% within 24–48 h; 7. CRP, LDH, or D‐dimer levels significantly increased. With respect to imaging manifestations (1), the following three points should be emphasized: possible concurrent plastic bronchitis or NP; significant elevation of CRP, LDH, and D‐dimer levels strongly suggesting the likelihood of NP and PE; and the likelihood of residual bronchiolitis obliterans. Children with imaging manifestations (2) often have allergic constitutions, frequently present with wheezing and shortness of breath, may have mixed infections, and can rapidly progress to respiratory failure. This is one of the reasons for ICU admission and poor response to mechanical ventilation and is prone to residual bronchiolitis obliterans. Currently, clinicians have relatively limited understanding of MP bronchiolitis, leading to misdiagnosis and underdiagnosis, necessitating increased awareness.

#### 2.3.3. Inclusion Criteria

(1) Meeting the above diagnostic criteria for MPP; (2) age < 14 years; (3) completing medical records; (4) and family members signing valid informed consent.

#### 2.3.4. Exclusion Criteria

(1) Co‐infection with other pathogens (bacteria or viruses); (2) congenital heart disease, immunodeficiency, or major organ dysfunction; and (3) incomplete clinical data.

#### 2.3.5. Grouping Criteria

SMPP: This refers to MPP that meets the diagnostic criteria for severe CAP and/or has a risk of long‐term pulmonary complications.

General MPP: Children who meet the diagnostic criteria for MPP but do not meet the criteria for severe pneumonia.

### 2.4. Clinical Data Collection

In accordance with the disease diagnostic criteria, the enrolled children were divided into the SMPP group and the common MPP group. The collected variables included general information, clinical characteristics, laboratory indicators, imaging examinations, and complications. General information included the child’s age, sex, season of onset, mode of delivery, prematurity, and presence of intrauterine infection at birth. Clinical characteristics included peak fever (the highest axillary body temperature during the illness), fever duration (days of fever), cough duration, and clinical symptoms (cough, headache and dizziness, dry and sore throat, wheezing and shortness of breath, difficulty breathing, nausea and vomiting, abdominal pain and diarrhea, muscle aches, rash, convulsions). Laboratory indicators included C‐reactive protein levels, lactate dehydrogenase levels, D‐dimer levels, ferritin levels, neutrophil percentage, white blood cell count, procalcitonin level, serum amyloid A level, absolute lymphocyte count, absolute monocyte count, red blood cell count, platelet count, MP antibody titer or nucleic acid positivity, and drug resistance. Imaging examinations included lesion location, extent, pulmonary consolidation, and pleural effusion. Complications included atelectasis, pleural effusion, rash, myocarditis, PE, and other complications. It should be noted that, although the diagnostic criteria for severe MPP referenced in Section 2.3.2 highlight the potential occurrence of serious sequelae, such as plastic bronchitis and bronchiolitis obliterans, these conditions were not specifically collected as separate outcome variables in this study. Given the retrospective design and the fact that these complications typically manifest later in the disease course or require specific bronchoscopic/histological confirmation, their incidence was not systematically documented in the acute‐phase medical records used for data extraction. Consequently, they are implicitly included under “other complications” but could not be analyzed as independent risk markers or outcomes. Data on macrolide resistance gene mutations and detailed antibiotic treatment regimens were not collected in this study.

### 2.5. Statistical Analysis

The data were analyzed using SPSS 25.0 software. The measurement data are expressed as the mean ± standard deviation ($\overset{¯}{x}$ ± *s*). If the measurement data conformed to a normal distribution, an independent‐sample *t* test was used for intergroup comparisons; otherwise, the Kruskal‒Wallis nonparametric test was employed. Count data are expressed as the number of cases or percentages and were analyzed using the chi‐square test or Fisher’s exact probability method. Multivariate logistic regression analysis was used to identify risk factors for SMPP. Variables with *p* < 0.05 in univariate analysis were further selected using a forward stepwise (likelihood ratio) method to construct the final multivariable model, and only variables that remained significant were retained. Receiver operating characteristic (ROC) curves were constructed to evaluate the predictive value of risk factors for SMPP. Two‐sided tests were conducted with a significance level of *α* = 0.05. *p* < 0.05 was considered to indicate statistical significance.

## 3. Results

### 3.1. Analysis of General Data and Clinical Characteristics

Statistically significant differences were observed between the severe and nonsevere groups of MPP in terms of onset season, preterm birth, presence of intrauterine infection at birth, pulmonary consolidation, pleural effusion, duration of fever, duration of cough, high fever, wheezing and shortness of breath, and difficulty breathing (*p* < 0.05), as shown in Table 1.

**TABLE 1:** Comparison of general and clinical characteristics between severe and nonsevere MPP groups.

Clinical data	Severe group (*n* = 49)	Nonsevere group (*n* = 97)	Statistical value	*p*
Age (years)	6.80 ± 3.86	7.01 ± 3.68	0.134	0.714
Gender (cases)			0.033	0.857
Male	23	44		
Female	26	53		
Season of onset (cases)			7.935	0.047**^a^**
Spring	5	27		
Summer	10	23		
Autumn	18	21		
Winter	16	26		
Mode of delivery (cases)			0.768	0.381
Vaginal delivery	27	46		
Cesarean section	22	51		
Premature birth (cases)	24	17	15.948	**< 0.001**
Presence of intrauterine infection at birth (cases)	20	20	6.677	**0.010**
Lung consolidation (cases)	22	7	29.040	**< 0.001**
Pleural effusion (cases)	9	4	—	0.010**^b^**
Duration of fever (days)	8.55 ± 2.13	4.06 ± 1.01	91.444	**< 0.001**
Hospital stay (days)	8.92 ± 4.15	4.84 ± 1.49	34.116	**< 0.001**
High fever (cases)	39	17	53.039	**< 0.001**
Cough (cases)	45	90	—	> 0.999^b^
Headache and dizziness (cases)	10	18	0.073	0.788
Dry and sore throat (cases)	8	16	0.001	0.979
Wheezing and shortness of breath (cases)	34	24	27.100	**< 0.001**
Difficulty breathing (cases)	22	12	19.280	**< 0.001**
Nausea and vomiting (cases)	8	11	0.715	0.398
Abdominal pain and diarrhea (cases)	6	10	0.125	0.724
Muscle soreness (cases)	10	19	0.014	0.907
Rash (cases)	4	2	—	0.098^b^
Convulsions (cases)	2	1	—	0.261^b^

*Note:* For categorical variables with two or more subcategories (e.g., gender, mode of delivery, and season of onset), the *p* values represent the overall comparison between the severe and nonsevere groups, using the chi‐square test or Fisher’s exact test as appropriate. Bold *p* values indicate statistical significance (*p* < 0.05).

^a^Overall *p* value for comparison among the four seasons.

^b^Fisher’s exact test.

### 3.2. Laboratory Test Indicators

Statistically significant differences in indicators, such as C‐reactive protein levels, lactate dehydrogenase levels, D‐dimer levels, ferritin levels, neutrophil percentage, and serum amyloid A levels, were detected between the severe and nonsevere groups (*p* < 0.05), as shown in Table 2.

**TABLE 2:** Comparison of laboratory indicators between severe and nonsevere MPP groups.

Laboratory indicators	Severe group (*n* = 49)	Nonsevere group (*n* = 97)	Statistical value	*p*
C‐reactive protein (mg/L)	15.25 ± 5.24	8.96 ± 1.97	8.103	**< 0.001**
Lactate dehydrogenase (U/L)	448.58 ± 52.70	151.71 ± 28.05	36.879	**< 0.001**
D‐dimer (mg/L)	3.01 ± 0.96	0.31 ± 0.10	19.726	**< 0.001**
Ferritin levels (μg/L)	347.14 ± 72.08	96.97 ± 28.71	23.377	**< 0.001**
Neutrophil percentage (%)	75.02 ± 8.49	49.55 ± 10.01	15.253	**< 0.001**
White blood cell count (× 10^9^/L)	7.67 ± 2.32	7.84 ± 2.41	0.163	0.686
Procalcitonin (ng/mL)	1.51 ± 0.49	1.43 ± 0.49	0.943	0.347
Serum amyloid A (mg/L)	231.30 ± 87.80	78.25 ± 36.04	11.714	**< 0.001**
Absolute lymphocyte count (× 10^9^/L)	1.94 ± 0.45	2.08 ± 0.51	−1.594	0.113
Absolute monocyte count (× 10^9^/L)	0.59 ± 0.09	0.55 ± 0.11	1.913	0.058
Red blood cell count (× 10^12^/L)	4.57 ± 0.58	4.56 ± 0.51	0.061	0.952
Platelet count (× 10^9^/L)	305.18 ± 49.03	297.20 ± 50.63	0.908	0.365

*Note:* For normally distributed continuous variables, data are expressed as mean ± standard deviation, and the statistical values represent the *t* statistic from an independent‐samples *t* test. For non‐normally distributed continuous variables, data are expressed as mean ± standard deviation for descriptive purposes, and the statistical values represent the *Z* statistic from the Mann–Whitney *U* test. Bold *p* values indicate statistical significance (*p* < 0.05).

### 3.3. Multivariate Analysis of Progression to SMPP

When severe MPP was used as the dependent variable (no = 0, yes = 1) and variables with *p* < 0.05 in the univariate analysis were entered as independent variables, the logistic regression analysis results revealed that premature birth, pulmonary consolidation, high fever, wheezing and shortness of breath, and difficulty breathing were risk factors for SMPP (*p* < 0.05), as shown in Table 3.

**TABLE 3:** Multivariate logistic regression analysis of severe *Mycoplasma pneumoniae* pneumonia.

Variable	β value	SE	Wald value	*p*	OR value	95% CI
Premature birth	1.557	0.677	5.293	0.021	4.747	1.259 ∼ 17.891
Pulmonary consolidation	1.663	0.687	5.855	0.016	5.277	1.372 ∼ 20.301
High fever	3.242	0.679	22.782	< 0.001	25.597	6.760 ∼ 96.926
Wheezing and shortness of breath	1.933	0.676	8.174	0.004	6.913	1.837 ∼ 26.017
Difficulty breathing	1.790	0.723	6.125	0.013	5.990	1.451 ∼ 24.722

### 3.4. Predictive Performance of Individual Indicators and the Combined Multivariable Model

**FIGURE 1:**
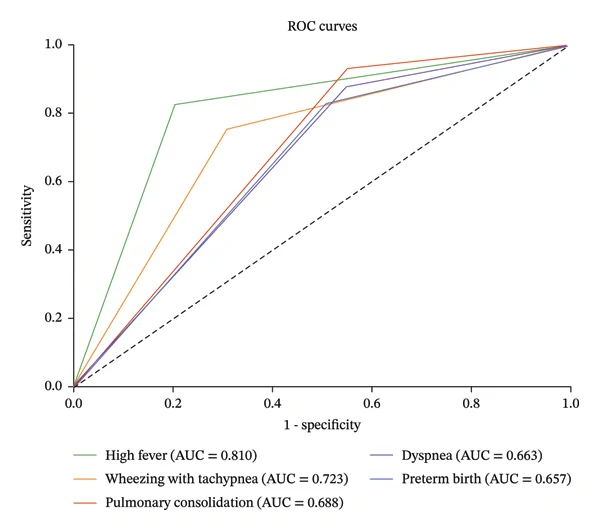
Receiver operating characteristic curves of five independent risk factors for predicting severe *Mycoplasma pneumoniae* pneumonia. The diagonal reference line indicates an AUC of 0.5. AUC: area under the curve.

To evaluate the discriminatory ability of the identified warning indicators for SMPP, ROC curve analysis was performed. The area under the curve (AUC), standard error (SE), 95% confidence interval (CI), and significance were calculated for each binary predictor. The results are presented in Table 4 and Figure 1.

**TABLE 4:** Receiver operating characteristic analysis of independent risk factors for severe *Mycoplasma pneumoniae* pneumonia.

Variable	AUC	SE	*p*	95% CI
Preterm birth	0.657	0.050	0.002	0.559–0.755
Pulmonary consolidation	0.688	0.050	< 0.001	0.590–0.787
High fever	0.810	0.040	< 0.001	0.732–0.889
Wheezing and shortness of breath	0.723	0.046	< 0.001	0.633–0.813
Difficulty breathing	0.663	0.050	0.001	0.564–0.761

Abbreviations: AUC, area under the curve; CI, confidence interval; SE, standard error.

High fever demonstrated the best single‐variable discriminatory performance, with an AUC of 0.810 (95% CI: 0.732–0.889, *p* < 0.001). Wheezing and shortness of breath yielded an AUC of 0.723 (95% CI: 0.633–0.813, *p* < 0.001), while pulmonary consolidation had an AUC of 0.688 (95% CI: 0.590–0.787, *p* < 0.001). Difficulty breathing and preterm birth showed relatively modest discrimination, with AUCs of 0.663 (95% CI: 0.564–0.761, *p* = 0.001) and 0.657 (95% CI: 0.559–0.755, *p* = 0.002), respectively. Although each individual risk factor alone provided only moderate discrimination, the combination of these clinical features contributed meaningfully to the early identification of children at risk for severe disease.

Although each of the above individual risk factors demonstrated moderate predictive value, clinical decision‐making typically requires a comprehensive assessment incorporating multiple variables. To further evaluate the overall performance of the constructed multivariable logistic regression model, we assessed its calibration, discriminative ability, and clinical classification accuracy. The results are summarized in Table 5.

**TABLE 5:** Goodness‐of‐fit and predictive performance of the multivariable logistic regression model for severe *Mycoplasma pneumoniae* pneumonia.

Performance metric	Value/estimate
Calibration	
Hosmer–Lemeshow test	*χ* ^2^ = 7.022, df = 8, ***p* = 0.534**
Explanatory power	
Nagelkerke *R* ^2^	**0.733**
Discriminative ability	
Combined AUC (from predicted probability)	**0.951** (95% CI: 0.916–0.985, *p* < 0.001)
Clinical utility (classification at cutoff = 0.5)	
Overall accuracy	**91.1%**
Sensitivity	**92.8%** (90/97)
Specificity	**87.8%** (43/49)
Positive predictive value	93.8% (90/96)^∗^
Negative predictive value	86.0% (43/50)^∗^

*Note:* Bold values indicate the main performance metrics of the model and do not imply statistical significance.

Abbreviations: AUC, area under the curve; CI, confidence interval; df, degrees of freedom; NPV, negative predictive value; PPV, positive predictive value.

^∗^PPV and NPV were derived from the same classification table at a cutoff of 0.5 and are provided as supporting descriptive statistics.

## 4. Discussion

This study retrospectively analyzed the clinical data of 146 children with MPP to systematically investigate the clinical features and risk factors of SMPP. We acknowledge that several candidate predictors identified in our multivariable model, including high fever, pulmonary consolidation, and wheezing, are themselves components of the SMPP diagnostic criteria referenced in the Chinese 2023 guidelines. This overlap—known as incorporation bias—means that these variables cannot be strictly interpreted as “independent prognostic predictors” or “causal risk factors” in the conventional epidemiological sense. However, the primary clinical utility of our analysis lies in evaluating the feasibility of using these baseline characteristics—all readily obtainable at admission—as early warning signals for bedside triage. While a patient presenting with persistent high fever and consolidation is already at the severe end of the clinical spectrum, our data suggest that the concurrent presence of multiple such features, particularly when combined with prematurity and respiratory distress, provides a practical and rapid screening tool to guide the intensity of monitoring and early intervention.

In univariate analysis, the SMPP group had significantly longer duration of fever, days of coughing, and hospital stays compared to the nonsevere group, and were more likely to present with lung consolidation, pleural effusion, high fever, wheezing and shortness of breath, and difficulty breathing. Regarding laboratory indicators, the SMPP group showed significantly elevated levels of C‐reactive protein, lactate dehydrogenase, D‐dimer, ferritin, neutrophil proportion, and serum amyloid A, suggesting a stronger inflammatory response and tissue damage in severe cases.

Multivariate logistic regression analysis further identified that preterm birth (OR = 4.747), pulmonary consolidation (OR = 5.277), high fever (OR = 25.597), wheezing and shortness of breath (OR = 6.913), and difficulty breathing (OR = 5.990) were significantly associated with SMPP in our cohort. First, preterm birth is among the significant risk factors for severe MPP. Preterm infants have immature lung development, insufficient alveolar counts, low pulmonary surfactant levels, and underdeveloped immune systems with deficiencies in both cellular and humoral immunity [16–18]. Therefore, preterm infants are more prone to disease progression after MP infection, which manifests as aggravated clinical symptoms and increased complications. Previous studies have also shown that preterm birth is closely related to the severity of respiratory infections, and the findings of this study are consistent with these findings, further emphasizing the importance of clinical follow‐up and intervention for respiratory infections in preterm infants. Second, the imaging manifestation of pulmonary consolidation is significantly associated with severe MPP.

In a prospective study of 1401 children with MPP, Huang et al. [19] reported that consolidation/atelectasis was the most severe imaging type, with markedly higher rates of refractory MPP (55.1% vs. 6.9%), NP (5.1% vs. 0.1%), and prolonged fever and hospitalization compared with bronchopneumonia. Pulmonary consolidation reflects extensive alveolar inflammatory reactions and large lesion involvement, indicating more severe inflammatory responses and immune damage [20–22]. Some studies have reported that children with larger areas of pulmonary consolidation often experience prolonged disease courses and extended hospital stays and are prone to concurrent pulmonary function impairment. The results of this study further corroborate this view. Pulmonary consolidation not only reflects the severity of pulmonary inflammation but is also closely related to abnormal T‐cell subsets, excessive inflammatory cytokine responses, and an increased risk of acute respiratory distress syndrome (ARDS) [23–25]. Imaging features, such as bilateral lung involvement or diffuse consolidation, have been confirmed to be important predictors of disease severity. These findings suggest that clinicians should consider pulmonary consolidation as a key reference indicator in imaging assessments of severity [26]. High fever, particularly a persistent body temperature ≥ 39°C, is also a risk factor for severe MPP. Persistent high fever is not only a clinical manifestation of intense inflammatory reactions but may also indicate an excessive immune response to pathogens, leading to a “cytokine storm” or immune damage [23]. Studies have shown that children with a fever duration of more than 7 days are more likely to develop refractory MPP and may experience complications, such as pleural effusion or extrapulmonary manifestations [27]. Therefore, for children with persistent high fever, clinicians should promptly assess their risk of progressing to severe disease and adjust anti‐infective and immunomodulatory treatment strategies as needed. With respect to respiratory symptoms, wheezing and shortness of breath and difficulty breathing have been confirmed as risk factors for severe MPP. This study revealed that these two clinical manifestations are significantly associated with disease severity, indicating that affected children experience significant airway obstruction, restricted pulmonary ventilation, and hypoxemia during infection. Previous studies have reported that wheezing and difficulty breathing often suggest airway hyperresponsiveness or diffuse inflammatory reactions. If not promptly corrected, these conditions can lead to acute respiratory failure and even life‐threatening complications [28–30]. Therefore, for children who exhibit marked wheezing and shortness of breath or difficulty breathing, respiratory function monitoring should be intensified, and respiratory support should be provided when necessary.

On the basis of the findings of this study, preterm birth, pulmonary consolidation, high fever, wheezing and shortness of breath, and difficulty breathing reflect the patient’s baseline condition, intensity of the inflammatory response, and degree of respiratory system damage to some extent, all of which can serve as important reference indicators for clinically assessing the risk of severe MPP. Notably, most of these risk factors can be identified at the time of hospital admission, suggesting their significant clinical value in the early identification of high‐risk patients and in guiding individualized treatment and prognostic evaluation. It should also be recognized that macrolide resistance is an important factor influencing disease progression. A recent systematic review and meta‐analysis encompassing 65 studies and 20,141 children demonstrated that macrolide‐resistant MPP is significantly associated with prolonged fever duration, extended hospitalization, higher proportions of severe and refractory cases, and increased pulmonary and extrapulmonary complications [11]. These findings underscore that, in addition to the clinical and imaging risk factors identified in this study, macrolide resistance may further amplify the risk of deterioration, reinforcing the need for early etiological diagnosis and appropriate antibiotic stewardship in high‐risk children.

This study has certain limitations. First, as this was a single‐center retrospective study with a limited sample size, selection bias may have occurred. Second, some laboratory indicators (such as inflammatory cytokine levels and drug resistance genes) were not included in the analysis, which might affect the comprehensive assessment of risk factors. Third, in this study, children with co‐infections were strictly excluded to construct a homogeneous cohort and isolate the disease mechanisms attributable solely to MP. This design choice, while enhancing internal validity, limits the real‐world applicability of our findings, as mixed infections are common in clinical practice and can act as critical effect modifiers. In real‐world settings, co‐infecting pathogens can synergistically amplify the inflammatory cascade, potentially lowering the threshold for severe disease development and altering the predictive utility of our identified risk factors. Consequently, a febrile child with atelectasis might be misclassified as low‐risk by our model if their deterioration is actually driven by an undetected co‐infection. Future pragmatic studies should stratify patients by co‐infection status to validate and refine the performance of our identified risk factors in a more heterogeneous population. Fourth, data on macrolide resistance and antibiotic treatment outcomes were not collected, which precluded analysis of how drug resistance or therapeutic regimens might influence disease severity. Future research should further explore the mechanisms and predictive models for MPP severity by incorporating multicenter, large‐sample studies along with molecular and immunological biomarkers, as well as macrolide resistance testing and treatment response data.

## 5. Conclusion

In conclusion, while causality cannot be established due to inherent incorporation bias, our study underscores the clinical value of readily available admission indicators. Children with SMPP exhibit significantly prolonged fever and cough, more severe clinical symptoms, and markedly elevated inflammatory markers. Prematurity, pulmonary consolidation, high fever, wheezing, and difficulty breathing are easily accessible baseline warning indicators that are strongly associated with disease progression. The combined model incorporating these five variables demonstrated excellent discriminative performance (AUC = 0.951) and high clinical accuracy (91.1%), supporting its utility as a pragmatic risk‐stratification tool at initial presentation. Strengthening early recognition and proactive monitoring in children presenting with these factors may facilitate timely escalation of care and potentially mitigate the risk of disease deterioration.

## Author Contributions

All listed authors actively participated in the study and meet the criteria for authorship. Chao Fu and Yunrong Li were responsible for the conception and design of the study. Xiang He, Chengcheng Li, and Shirong Luo were responsible for the acquisition and organization of clinical, laboratory, and imaging data from electronic medical records. Chao Fu, Xiang He, and Chengcheng Li were responsible for the statistical analysis using SPSS 25.0 and the interpretation of the results. Chao Fu and Shirong Luo were responsible for drafting the manuscript. Yunrong Li, Xiang He, and Chengcheng Li were responsible for critically revising the manuscript for important intellectual content. Chao Fu, Xiang He, Chengcheng Li, Shirong Luo, and Yunrong Li contributed equally to the study.

## Funding

This work was supported by the Science and Technology Fund of Guizhou Provincial Health Commission (gzwkj2024‐429).

## Disclosure

All authors read and approved the final manuscript and agreed to be accountable for all aspects of the work.

## Conflicts of Interest

The authors declare no conflicts of interest.

## Data Availability

The data that support the findings of this study are available from the corresponding author with the permission of the First People’s Hospital of Zunyi (The Third Affiliated Hospital of Zunyi Medical University).
